# Precision Medicine Tools to Guide Therapy and Monitor Response to Treatment in a HER-2+ Gastric Cancer Patient: Case Report

**DOI:** 10.3389/fonc.2019.00698

**Published:** 2019-08-06

**Authors:** Adriana Aguilar-Mahecha, Sarah Joseph, Luca Cavallone, Marguerite Buchanan, Urszula Krzemien, Gerald Batist, Mark Basik

**Affiliations:** ^1^Department of Oncology, Lady Davis Institute, McGill University, Montreal, QC, Canada; ^2^Segal Cancer Center, Jewish General Hospital, Montreal, QC, Canada; ^3^Department of Surgery, Jewish General Hospital, Montreal, QC, Canada

**Keywords:** precision medicine, patient derived xenograft, ctDNA, gastric cancer, HER-2+, T-DM1

## Abstract

Trastuzumab, has played a major role in improving treatment outcomes in HER-2 positive gastric cancer. However, once there is disease progression there is a paucity of evidence for second line therapy. Patient-derived xenografts (PDXs) in combination with liquid biopsies can help guide individual therapeutic decisions and have now started to be studied. In the present case we established a PDX model from a metastatic HER-2+ gastric cancer patient and after the first engraftment passage we performed a mouse clinical trial to test T-DM1 as an alternative therapy for the patient. The PDX tumor response served as a guide to administer T-DM1 therapy to the patient who responded to treatment before relapsing 6 months later. Throughout out the clinical follow up of the patient, ctDNA levels of HER-2 copy number and a PIK3CA mutation were monitored and we found their correlation with drug response and disease progression to outperform that of CEA levels. This study highlights the utility of applying precision medicine tools combining PDX models to guide therapy with circulating tumor DNA (ctDNA) to monitor treatment response and disease progression.

## Background

Precision Medicine focuses on tailoring treatment for the tumor specific characteristics of individual patients (1). This implies moving beyond using large phase 3 trials of unselected, though superficially similar patients, to select the best therapy for one patient. The potential is enormous both for patients and for the healthcare system. Important clinical and biological challenges still stand in the way, including the fact that intra-tumoral heterogeneity may limit the ability of tumor biopsies to capture the complete biological portrait of the tumor (2). Moreover, next generation sequencing frequently identifies multiple variants, sometimes more than one that can be matched to an available drug, and distinguishing driver from passenger variants remains elusive (3, 4). Efforts are underway to address this latter point by including transcriptomic and proteomic analyses (5–7), but a more patient-focused and direct approach is presented here. An attractive solution to the dearth of actionable mutations is to directly test tumor response on patient- derived tissue samples. Patient-derived xenografts (PDX) have provided new insights to efficiently test clinical samples with various drug combinations. This allows for correlations between PDX tumor response and patient clinical response (8). Although the use of PDX models to guide therapeutic decision has been challenging because of the lengthy process of tumor engraftment, expansion, and drug testing (9), this approach has been studied in many solid tumors such as breast (10), colon (11), renal cell (12), and duodenal cancer (13). Another possible solution to the problem of intra-tumor heterogeneity is the use of circulating tumor DNA (ctDNA), which may be a broader method to assess tumor molecular composition (14). We present a case of a patient with metastatic human epidermal growth factor receptor (HER-2) positive gastric carcinoma in which treatment decisions were guided by PDX data and treatment response was monitored with ctDNA markers on serial blood samples. This is a prime example of the power of cutting-edge personalized precision medicine based on PDX testing and ctDNA measurements in changing clinical care in oncology.

## Case Presentation

**Figure 1:**
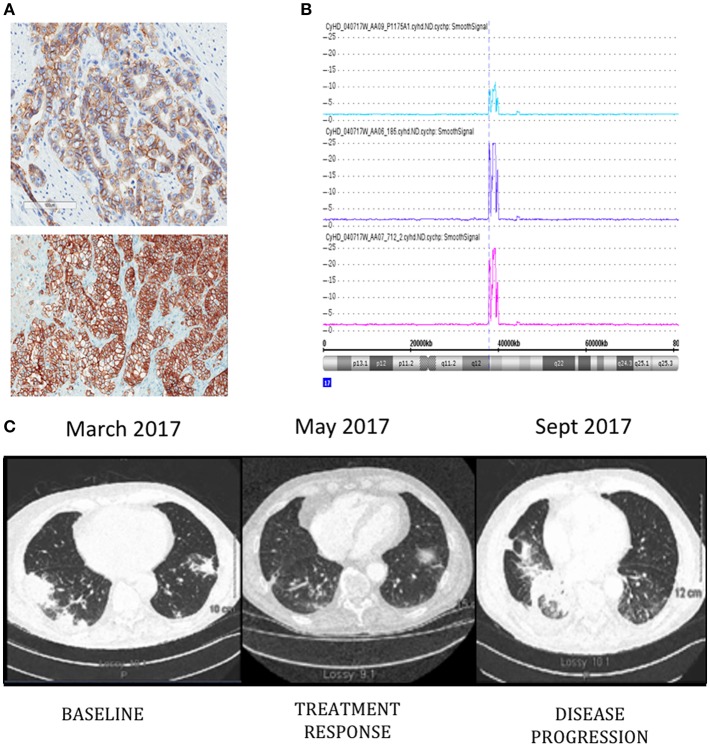
ERBB2 expression detected by immunohistochemistry **(A)** in original patient tumor (top) and PDX 712 tumor (bottom). Visualization of Chromosome 17 region containing the ERBB2 amplicon **(B)**, the number of ERRB2 copies are shown in original patient tumor (blue) and PDX tumors F0 (purple) and F1 (pink). Computed tomography (CT) images of the patient's thorax **(C)** before T-DM1 treatment (left panel), at the time of treatment response (middle panel), and progression (right panel).

Patient is a 76-year-old male diagnosed with metastatic HER-2 positive moderately differentiated gastric adenocarcinoma in April 2011. Staging scans demonstrated metastatic lesions in the lung and liver at time of diagnosis. The patient was started on carboplatin and paclitaxel followed by Xeloda along with Trastuzumab. He had a dramatic response, with disappearance of all evidence of disease, except for modest PET uptake in the gastric primary site. Since he was asymptomatic, he was maintained on single agent Trastuzumab from June 2012–2016. Serial surveillance PET CT scans demonstrated no FDG-avid disease up until he began to experience increasing symptoms of post-prandial dysphagia and epigastric pain in 2015. In July 2015, a gastroscopy showed a large ulcerative lesion in the lesser curvature of the stomach, which was significantly larger than on previous examinations. In December 2015 he underwent open subtotal gastrectomy with a Roux-en-Y anastomosis. Pathology confirmed the same intestinal type adenocarcinoma G3 poorly differentiated, pT4pN3b, with 27 out of 35 lymph nodes involved. HER-2 status was positive by immunohistochemistry (IHC) and copy number analysis using Cytoscan HD (Figures 1A,B). A piece of this tumor was collected for molecular analysis and PDX engraftment for drug testing. In February 2016, he developed melena, and was found to have a bleeding ulcer, which was biopsy proven invasive adenocarcinoma. He underwent palliative radiation therapy with concurrent low dose Capecitabine chemotherapy. In August 2016, new hypermetabolic lung nodules were detected on another PET scan. Capecitabine was discontinued in November 2016 and a CT scan performed in December 2016 showed the appearance of new sub-pleural hypermetabolic nodules. By this time, PDX results showed excellent response of PDXs to T-DM1. We had difficulty in obtaining off-label T-DM1, based on the negative results of a randomized study of T-DM1 in this setting (15). Nevertheless, since there was no obvious alternative, we persisted, and he finally began treatment with T-DM1 in March 2017. The patient did not receive any medication between November 2016 and the beginning of T-DM1. The patient completed 3 cycles of treatment and a CT scan in early May 2017 demonstrated treatment response and the patient reported complete disappearance of coughing symptoms and felt more ease at breathing (Figure 1C). T-DM1 was interrupted for 3 weeks to confirm that the imaging was indeed demonstrating tumor and not infection in the lung and treatment resumed in July 2017. The patient was maintained on T-DM1 with no reported side-effects until disease progression in September 2017 (Figure 1C) and he passed away from CNS disease in January of 2018.

## PDX Development and Drug Testing

**Figure 2:**
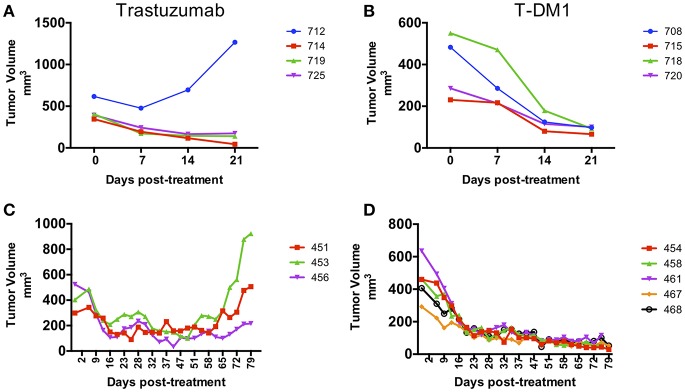
Response to treatment in PDX models. PDX drug efficacy of Trastuzumab and T-DM1 in two consecutive studies performed on NOG mice engrafted with F0 PDX tumor **(A,B)** and with Trastuzumab resistant PDX tumor from mouse 712 (F1) **(C,D)**. Data are presented as tumor volume (mm^3^) during treatment days (x axis). Each colored line represents a single mouse PDX.

Pieces of tumor from the gastrectomy were engrafted subcutaneously in one immune-deficient mouse (NOG). Adenocarcinoma was confirmed by histology in the PDX tumor and HER-2 amplification was confirmed by copy number analysis and IHC and matched the original patient tumor (Figures 1A,B). An initial drug study was performed at the first PDX generation passage (F1) in May 2016. Interestingly, of the 4 mice treated with Trastuzumab, only 1 demonstrated drug resistance while treatment with T-DM1 resulted in tumor regression in all mice tested (Figures 2A,B). A second drug study was performed in October 2016 on PDXs (F3) generated from the Trastuzumab resistant PDX (mouse 712). This study showed emergence of resistance to Trastuzumab and validated the rapid and durable response to T-DM1 (Figures 2C,D).

## Development of Personalized ctDNA Assays and Monitoring of Response to Treatment

**Figure 3:**
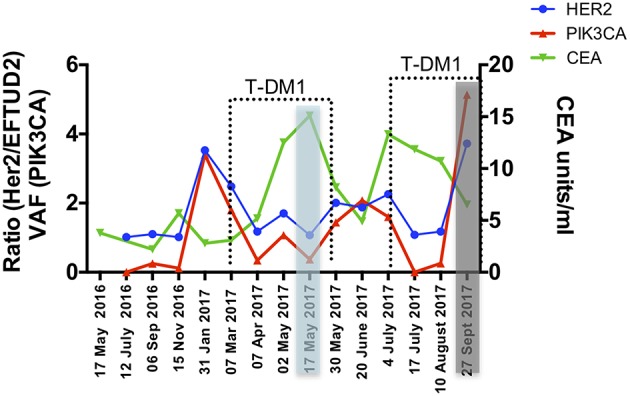
Circulating markers were measured in serial plasma collected from the patient throughout 18 months of clinical management. Data are presented as variant allele frequency (VAF) for the PIK3CA mutation, ratio of number of copies of HER-2 /EFTUD2 and units per ml for CEA. Dashed lines delineate time points of T-DM1 treatment and shaded areas correspond to clinical response (blue) and progression (gray).

The gastrectomy tumor and PDX tissues underwent targeted MiSeq for the analysis of somatic mutations and copy number analysis. Four different somatic mutations were identified (Table 1) and digital droplet PCR (ddPCR) assays were developed for the PIK3CA variant and the HER-2 amplification. ddPCR analyses were performed on serial bloods collected at each clinical follow up. We observed an increase in ctDNA levels in January 2017 as resistance to Trastuzumab was setting in clinically, with new lesions appearing in the lungs. We noted that ctDNA levels had already begun to fall significantly in April 2017, 3 weeks after starting T-DM1 treatment. Interestingly this fall in ctDNA levels of both HER-2 copy numbers and mutated PI3KCA correlated with clinical response while CEA levels were markedly increasing (Figure 3). After a short interruption in T-DM1, a second cycle was given, not before some rise in ctDNA levels. The administration of this second T-DM1 cycle resulted in a prompt decrease in ctDNA levels, until progression, which occurred in September 2017 (Figure 1C). At this point, ctDNA levels had markedly risen, coincident with disease progression, while, in contrast CEA levels had decreased and did not reflect disease status (Figure 3).

**Table 1:** Somatic mutations identified in original patient tumor engrafted in PDX (F0) and the Trastuzumab resistant PDX 712 (F1).

**Gene**	**Variant**	**Allele frequency F0**	**Allele frequency F1 (712)**
PIK3CA	c.317G>T	0.44	0.35
PIK3R1	c.428-21G>A	0.99	0.99
CDHI	c.687+1_687+2delGT	0.93	0.93
TP53	c.733G>A	0.99	0.99

## Discussion

The era of Precision Medicine is highlighting new technologies that help identify and predict therapies that may benefit patients, in the absence of phase 3 supportive data for an unselected but similar patient group. In this case, the PDX model predicted response to T-DM1 in second line for HER-2+ gastric cancer, despite negative phase 3 data in similar but otherwise unselected patients. Our case highlights how patient treatment planning can be tailored based on PDX models and how treatment response can be monitored with ctDNA. Even after HER-2 functional blockade with Trastuzumab was no longer clinically effective, the persistence of HER-2 in the tumor served both as an effective passive target for the antibody-dependent cytotoxic approach, as well as a tumor-related cell-free DNA biomarker that appears to be significantly more effective than current standard blood CEA test in predicting disease status. The ability to quantitate ctDNA over time has tremendous implications both for clinical progress of tumor volume and the biological study of tumor evolution over its entire trajectory, while avoiding serial tumor biopsies that are often difficult for patients to accept (16, 17). In our case, a PDX was generated from the gastric cancer as it became resistant to Trastuzumab. Interestingly, Trastuzumab resistance was observed in only one of 4 mice initially tested, highlighting the heterogeneity of PDX samples with respect to treatment response. Further testing with Trastuzumab of PDXs generated from the Trastuzumab resistant mouse showed the emergence of resistance after 2 months of treatment suggesting an enrichment for drug resistant clones. This highlights the limitations of using a single PDX mouse for treatment decisions. On the other hand, response to T-DM1 was uniform in all PDX mice tested. Ideally, to minimize the effects of genomic drift on drug response, drug testing should be performed at early PDX passages. Here we performed drug studies at passages F1 and F3 with similar responses to T-DM1. Although we obtained PDX evidence of T-DM1 response in May 2016, the patient started T-DM1 treatment in March 2017 after a long process to access T-DM1 off label. Interestingly, the PDX model established from the primary gastric tumor could predict patient response of the lung metastatic lesions, and this, months later, and despite the tumor evolution that could have taken place after capecitabine treatment. Although many reports have correlated clinical drug response with response in PDXs, the majority of these studies have done so in a retrospective manner (8, 18–21). The use of PDXs to guide clinical treatment decisions in a prospective manner or in real-time is challenging and very few cases have been published (22–24). Our study demonstrates the feasibility of using PDXs to identify personalized effective therapies in patients with limited therapeutic options. Circulating tumor DNA (ctDNA) is an emerging method to monitor treatment response oftentimes in advance of imaging evidence. The value of ctDNA testing in gastric cancer patients has been studied using targeted panels or specific ddPCR assays to detect somatic mutations and gene amplifications (25–27). In particular, the measurement of HER-2 copy number levels in ctDNA holds great promise for its potential clinical use to identify gastric cancer patients who may benefit from Trastuzumab and to monitor response to treatment and disease progression (27–29). In our study we used ctDNA assays to detect the HER-2 amplification and a PIK3CA variant in the peripheral blood and monitor drug response. We observed that changes in both ctDNA markers paralleled each other very tightly and that ctDNA levels were sensitive predictors of response and disease progression and outperformed CEA testing. These findings are consistent with other reports in which increase in ctDNA levels coincide with recurrence of gastric cancer (14, 28, 29) and those of Garcia-Murillas et al. (30), in which ctDNA level changes anticipated imaging evidence of tumor progression by almost 8 months. Our case illustrates the direction that cancer care is evolving toward. We created PDX models that guided our patient's care, and followed tumor response using ctDNA, based on sequencing of the tumor. This case highlights the power of combining 2 cutting edge tools, PDXs and ctDNA, which together allow us to more successfully apply precision medicine in metastatic gastric cancer.

## Methods

(For a detailed description of the methods, see Supplementary Methods).

### Ethics

The patient provided written informed consent for sample collection and use as part of the JGH Central Biobank, the biobank protocol (#10–153) was approved by the Jewish General Hospital research ethics committee. We have obtained written informed consent from a participant's relative for the publication of this case report and any potentially-identifying images/information.

### PDX Development

Two pieces (2 × 2 mm) of tumor were implanted sub-cutaneously into both flanks of one 5-week old NOG (NOD.Cg-Prkdcscid Il2rgtm1Sug/JicTac) mouse (Taconic Labs). Tumor growth was monitored with calipers and 3 months later, when the tumor reached 1,500 mm^3^, fresh pieces of tissue were engrafted into one flank of 12 NOG mice. Mice were treated with vehicle saline, Trastuzumab (5 mg/kg, i.p once a week) or T-DM1 (10 mg/kg, i.p, once a week). A second drug study was carried out using the trastuzumab resistant PDX mouse 712 which was transferred into both flanks of 3 NOG mice. Pieces of tumors from 2 of these mice were used to generate the next treatment cohort of 11 NOG mice which were treated with the same drugs as above. Tumor size was monitored by electronic caliper measurement and mice were monitored for signs of toxicity (weight loss, bleeding etc) and tumor burden.

Nucleic acid extractions: DNA and RNA were extracted from the patient's tumor and PDX samples as previously reported (31).

### ctDNA Assays

Cell free DNA (cfDNA) extraction and development of ddPCR assays were performed as previously reported (32). Briefly, in order to estimate PIK3CA (G106V) AF a pre-amplification of 5–10 ng cfDNA step was incorporated prior to ddPCR reaction using primers listed in Supplementary Table S1a. The PCR amplified products were diluted and combined with mutant and wild-type probes for PIK3CA G106V mutation detection (Supplementary Table S1a). ddPCR was then performed on Biorad C1000 thermal cycler incubating the plates at 95°C for 10 min followed by 40 cycles of 95°C for 15 s, 55°C for 60 s, followed by 10 min incubation at 98°C and plates were read on a Bio-Rad QX200 droplet reader.

To test HER-2 gene amplification status with ddPCR we used the EFTUD2 gene as a reference since it shows a highly stable copy number ratio with the ERBB2 locus (33). HER-2 amplification was defined as the HER-2/EFTUD2 ratio. For each sample 10–50 ng of cfDNA were partitioned into 20,000 droplets with primers and probes for HER-2 and EFTUD2 genes (Supplementary Table S1b). ddPCR was then performed on Biorad C1000 thermal cycler incubating the plates at 95°C for 10 min followed by 40 cycles of 98°C for 15 s, 55°C for 60 s, followed by 10 min incubation at 98°C and plates were read on a Bio-Rad QX200 droplet reader.

## Data Availability

Raw data were generated at the Sick Kids Hospital and Jewish General Hospital Molecular Pathology facilities. Derived data supporting the findings of this study are available from the corresponding author upon request.

## Ethics Statement

This study was carried out in accordance with the recommendations of the Jewish General Hospital Ethics board with written informed consent from all subjects. The patients gave written informed consent in accordance with the Declaration of Helsinki. The protocol was approved by the Jewish General Hospital Ethics Review Board.

## Author Contributions

AA-M, MBa, and GB designed the study. SJ and UK collected clinical data. UK collected clinical samples, MBu performed PDX experiments, LC developed ctDNA assays. AA-M supervised and managed the data generation and data analysis. MBa, GB, AA-M, and LC contributed to the analysis and interpretation of the data. AA-M, MBu, MBa, GB, SJ, and LC reviewed all drafts of the manuscript.

### Conflict of Interest Statement

The authors declare that the research was conducted in the absence of any commercial or financial relationships that could be construed as a potential conflict of interest.
