# Supplementary material for: Precision Medicine Tools to Guide Therapy and Monitor Response to Treatment in a HER-2+ Gastric Cancer Patient: Case Report
Source: Front Oncol. 2019 Aug 6;9:698. doi: 10.3389/fonc.2019.00698 (PMC6691136; doi:10.3389/fonc.2019.00698)
Supplement: Supplementary file 1 [file Data_Sheet_1.PDF]

Supplementary Material to:

Precision medicine tools to guide therapy and monitor response to treatment in a Her2+ gastric cancer patient: Case report

Adriana Aguilar-Mahecha[1], Luca Cavallone[1], Sarah Joseph[2], Marguerite Buchanan[1], Urszula Krzemien[1], Gerald Batist [1,2], Mark Basik [1,3]

[1] Lady Davis Institute, Department of Oncology, McGill University, Montreal, Quebec, Canada

[2]Jewish General Hospital, Department of Surgery, Montreal, Quebec, Canada

[3]Jewish General Hospital, Department of Oncology, Montreal, Quebec, Canada

Supplementary Methods:

Cytoscan HD analysis: Copy number analysis was performed using CytoScan-HD arrays (Affymetrix) at the Centre for Applied Genomics (Sick Kids Hospital, Toronto) following the manufacturer's protocol (1). The ERRB2 amplification was confirmed using Affymetrix Chromosome Analysis Suite (ChAS).

Immunohistochemistry: IHC was performed at the Segal Cancer Centre Research Pathology Facility (Jewish General Hospital). Tissue samples were cut at 4-µm, placed on SuperFrost/Plus slides (Fisher) and dried overnight at 37°C, before IHC processing. The slides were then loaded onto the Discovery XT Autostainer (Ventana Medical System). All solutions used for automated immunohistochemistry were from Ventana Medical System (ROCHE) unless otherwise specified. Slides underwent de-paraffinization, heat-induced epitope retrieval (CC1 prediluted solution Ref: 950-124, standard protocol). Briefly, Rabbit monoclonal anti Her2 (clone 29D8) from Cell Signaling (Catalog nb#2165) diluted 1:100 was manually applied for 32min at 37°C then followed by the appropriate detection kit (OmniMap anti-Rabbit-HRP, Ref: 760-4311 and ChromoMap-DAB Ref: 760-159). A negative control was performed by the omission of the primary antibody. Slides were counterstained with Hematoxylin for 8 minutes, blued with Bluing Reagent for 8 minutes, removed from the autostainer, washed in warm soapy water, dehydrated through graded alcohols, cleared in xylene, and mounted with Eukitt Mounting Medium (EMS, Ref: 15320). Sections were analyzed by conventional light microscopy and scanned.

Sequencing: Targeted sequencing was performed at the Molecular Pathology Core (Jewish General Hospital) using a Nimblegen hybrid capture panel IRN4000020360 with SeqCap probes capturing in total 148634bp. The panel is described in supplementary Table S2. Sequencing was performed on a MiSeq (Illumina) using Miseq v2 chemistry reagent kits following the manufacturer's protocols.

References:

1. Uddin M, Thiruvahindrapuram B, Walker S, Wang Z, Hu P, Lamoureux S et al. A High-Resolution Copy Number Variation Resource for Clinical and Population Genetics. Genet Med. 2015 Sep; 17(9): 747–752.

Supplementary Table S1.

a) Primer and probes for PIK3CA

Gene	AA variation	SNV	Primer Forward	Primer Reverse	WT probes	MUT probes	Fragment bp	ddPCR temperature condition (℃)
PIK3CA	p.G106V	G>T	AGACGACTTTGTGACCTTCG	CCAATTTCTCGATTGAGGATCTTT	AG+TA+G+G+CAA+CC	AG+TA+G+T+CA+A+CCG	95	55

b) Primers and probes for HER2 and EFTUD2

Gene	Primer Forward	Primer Reverse	ddPCR probes	Fragment bp	ddPCR temperature condition (C)
HER2	ACAACCAAGTGAGGCAGGTC	GTATTGTTCAGCGGGTCTCC	CCCAGCTC+TTTG +AGG ACAAC	115	58
EFTUD2	GGTCTTGCCAGACACCAAAG	TGAGAGGACACACGCAAAAC	ACAT+C+CTTTGG+CTTTT+GA	118	58

Supplementary Table S2. Description of MiSeq gene panel

Gene	Exon
ABL1	4-7
AKT1	3
ALK	5, 9, 19-29
BRAF	11, 15
CALR	1-9
CDKN2A	1-3
CEBPA	1
CTNNB1	3
DNMT3A	21-23
EGFR	1-8, 15, 18-21
FGFR1	4, 5, 8 ,12-15
FGFR2	6, 7, 9, 11, 12
FGFR3	7-10, 14-18
FLT3	11, 14-16, 20
GNA11	4, 5
GNAQ	4, 5
HRAS	2-4
JAK2	11-16, 19-21
KIT	2, 9-11, 13-15, 17, 18
KRAS	2-4
MAP2K1	1-11
MET	2, 14, 16, 19
MPL	10
NF1	1-57
NMP1	4-6, 10, 11
NRAS	2-4
PDGFRA	11, 12, 14, 15, 18
PIK3CA	2, 5, 8, 10, 14, 21 (1,4,7,9,13,20)
PTEN	1-9
SMAD4	2-12
SRC	14
TP53	4-10
ARID1A	1-20
AFF2	1-21
CDH1	1-16
CDKN1B	1, 2
CTCF	3-12
ESR1	1-8
FLT4	1-30
FOXA1	1, 2
GATA1	2-6
JAK3	2-24
MAP2K4	1-11
MAP3K1	1-20
KMT2C	1-59
NCOR1	2-46
PAK7	4-11
PDGFRA	2-23
PIK3R1	1-16
PTPN22	1-21
PTPRD	12-43
RB1	1-27
RET	1-20
RUNX1	1-6
SPEN	1-15
TSC1	2-23
EZH2	16-18
KEAP1	2-6
STAT3	21
NRF1	2-11
IDH1	4
IDH2	3,4
STK11	1-8
BAP1	1-17
